# Supplementary material for: Liquid-microjet photoelectron spectroscopy of the green fluorescent protein chromophore
Source: Nat Commun. 2022 Jan 26;13:507. doi: 10.1038/s41467-022-28155-5 (PMC8791993; doi:10.1038/s41467-022-28155-5)
Supplement: Supplementary file 1 — Supplementary Information [file 41467_2022_28155_MOESM1_ESM.pdf]

Supplementary Information

Liquid-microjet photoelectron spectroscopy of the green fluorescent protein chromophore

Omri Tau,[†,‡] Alice Henley,[†,‡] Anton N. Boichenko,[¶] Nadezhda N. Kleshchina,[¶] River Riley,[†] Bingxing Wang,[†,‖] Danielle Winning,[†] Ross Lewin,[†] Ivan P. Parkin,[†] John M. Ward,[§] Helen C. Hailes,[†] Anastasia V. Bochenkova*,[¶] and Helen H. Fielding*,[†]

†*Department of Chemistry, University College London, 20 Gordon Street, London, WC1H 0AJ, U.K.*

‡*Contributed equally to this work*

¶*Department of Chemistry, Lomonosov Moscow State University, 119991 Moscow, Russia*

§*The Advanced Centre for Biochemical Engineering, Department of Biochemical Engineering, University College London, Gower Street, London, WC1E 6BT, U.K.*

‖*Current address: College of Chemistry and Chemical Engineering, Henan Institute of Science and Technology, Eastern Hualan Avenue, Xinxiang, 453003, China*

E-mail: bochenkova@phys.chem.msu.ru; h.h.fielding@ucl.ac.uk

*To whom correspondence should be addressed

Supplementary methods

Instrument transmission function and fit-sensitivity at low eKE

We have measured the relative photoelectron signal loss for low eKEs by recording non-resonant two-photon photoelectron spectra of NO at a range of wavelengths (237–250 nm). For each photoelectron spectrum, the peak intensities of each band were determined relative to the 0-0 vibronic band and their relative variation with eKE is then plotted in Supplementary Fig. 1. The transmission function decreases most significantly for eKE < 0.3 eV. We fit an exponential decay function through the data to approximate a continuous description of the instrument transmission function.

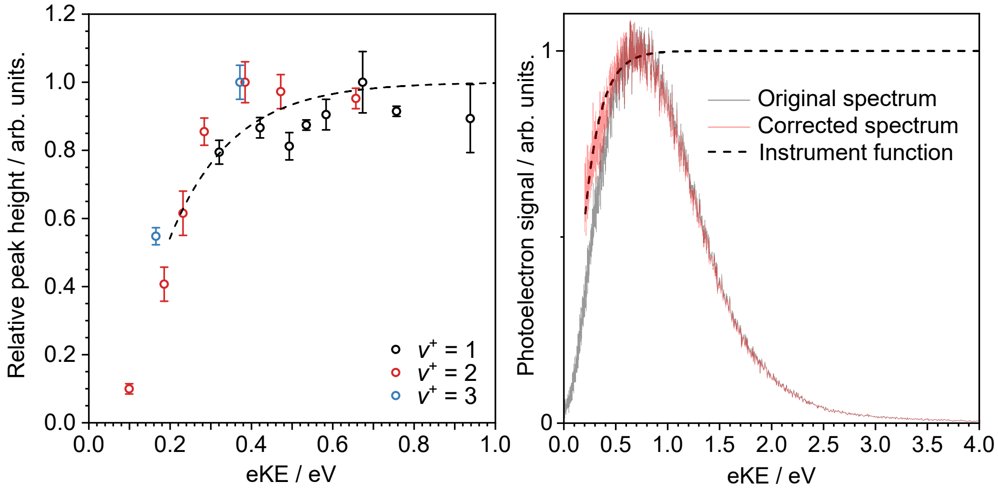

Supplementary Figure 1: Determination and application of the instrument transmission function. Left: Relative peak heights for a series of non-resonant two-photon photoelectron spectra of NO. The dashed line is an exponential fit to all data points and is plotted for eKE > 0.2 eV, since we have relatively few data points at lower eKEs. Error bars were calculated by taking a minimum/maximum gaussian fit to each feature in the photoelectron spectra of NO and taking the difference between the peak heights of the fits. Right: An example of the application of the instrument function correction for the 300 nm *p*-HBDI$^-$ spectrum, before correction for the vacuum level offset between the interaction region and the analyser, described in Absolute energies below. We only apply the instrument function for eKEs > 0.2 eV due to the uncertainty in its profile at lower eKEs.

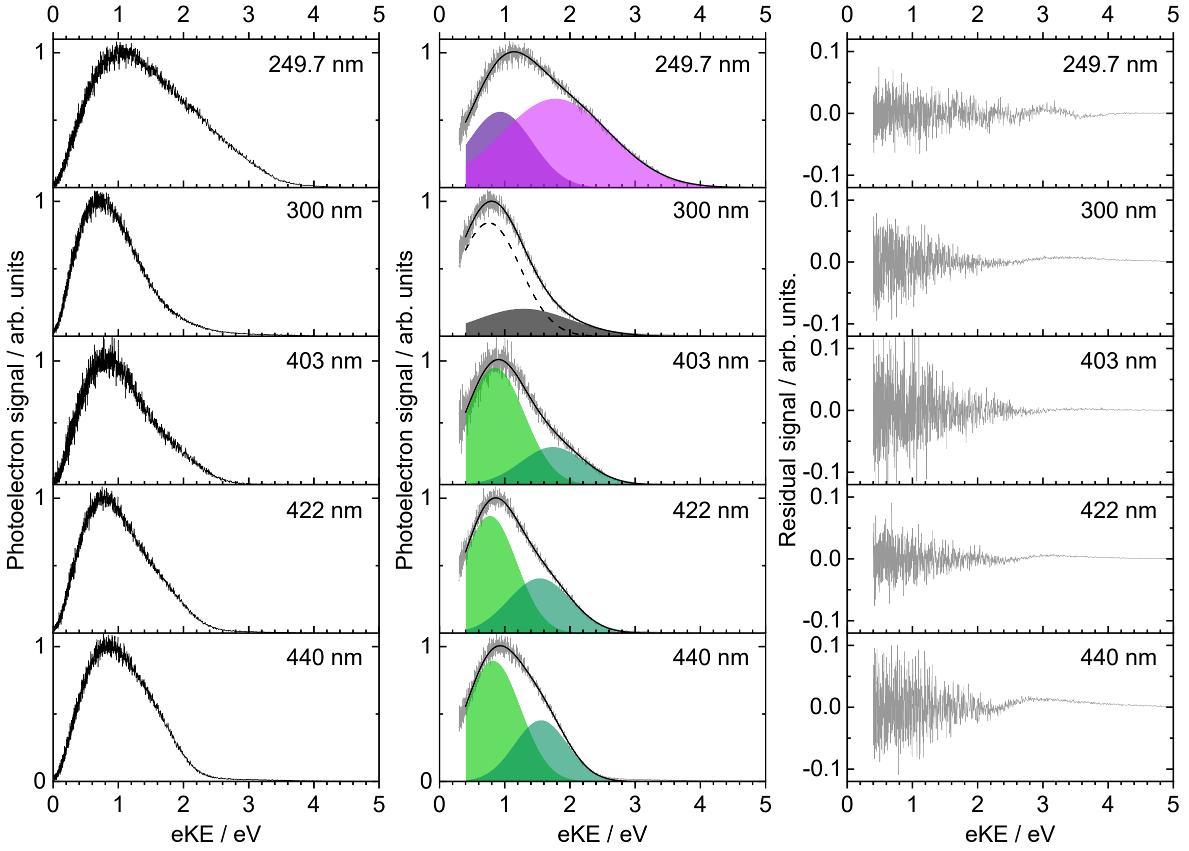

Supplementary Figure 2: Instrument function correction to the multiphoton detachment spectra of p-HBDI$^-_{aq}$. Left: Original MP detachment spectra of a 20 μM aqueous solution of p-HBDI$^-$ before correcting for the instrument function. Centre: Spectra corrected for the instrument transmission function and then shifted by +0.1 eV (grey lines) to account for the vacuum level offset between the interaction region and the analyser described in Absolute energies below, as shown in Fig. 2a in the main text. Gaussian fits described in the main text and in Supplementary Fig. 3 below are also shown. Right: Residuals from the Gaussian fits.

To investigate the robustness of the Gaussian fits at low eKE, we have fit spectra with low eKE cut-off limits of 0.2, 0.3 and 0.4 eV (Supplementary Fig. 3). These limits refer to the measured eKEs before the spectra are shifted by +0.1 eV to account for the vacuum level offset between the interaction region and the analyser (Absolute energies below), so the corresponding true eKE cut-off limits are 0.3, 0.4 and 0.5 eV. For each photodetachment feature, the centres of the fitted Gaussians agree within experimental error with those obtained with different fit conditions. Due to uncertainty in the instrument function correction for measured eKEs < 0.2 eV, we chose to be conservative and use the fits obtained with the measured 0.3 eV (true 0.4 eV) cutoff.

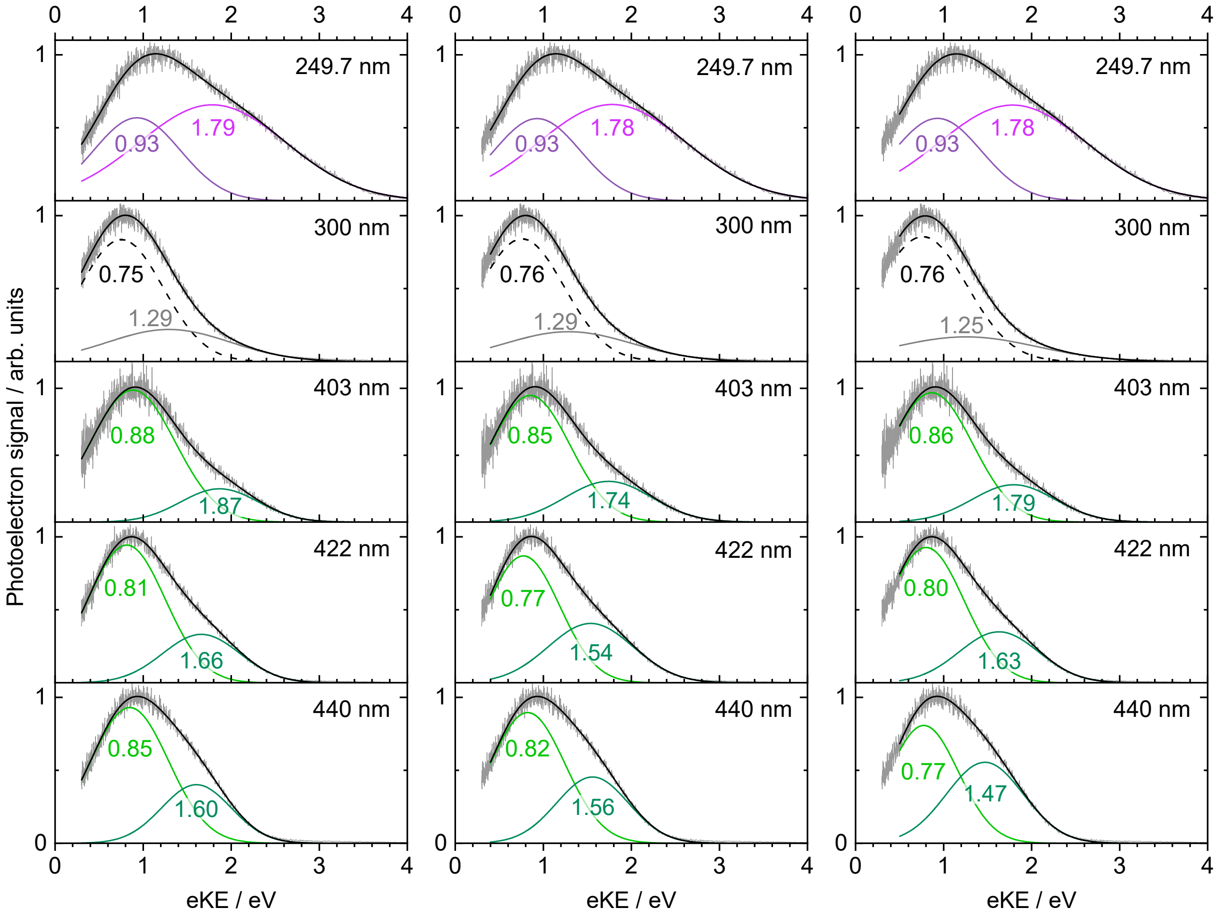

Supplementary Figure 3: Comparison of gaussian fits with differing cut-off limits. MP detachment spectra (grey lines) of aqueous *p*-HBDI^{-} plotted as a function of true eKE (after correcting for the vacuum level offset between the interaction region and the analyser) and fit with Gaussian line shapes (colours match those in Fig. 2) with true low eKE fit cut-offs of 0.3 eV (left), 0.4 eV (centre) and 0.5 eV (right). Gaussian centres obtained from the fits are indicated in the plots.

Absolute energies

When reporting absolute energies, it is necessary to calibrate the TOF accurately and account for both the streaming potential of the liquid-microjet and the difference between the vacuum level of the interaction region and the analyser.[1,2] We have accounted for these in our measurements using the methods described below.

Calibration of the time-of-flight (TOF) spectrometer was performed following procedures similar to those reported in Ref. 3. Briefly, photoelectron spectra following 2+1 REMPI of Xe at 249.7 nm and non-resonant MPI of NO with a series of wavelengths (237.0 – 266.5 nm) were recorded to obtain a set of time-of-flight measurements associated with well-defined eKEs.[4,5]

The streaming potential of a liquid-microjet can be controlled by adjusting the concentration of electrolyte salt used in solution, or the flow rate of the solution through the microjet. Recent work has also shown that a bias voltage can be applied to the solution to flatten the potential in the interaction region.[1,6] For the measurements reported here, we have flattened the potential in the interaction region of our spectrometer by coating the liquid nozzle holder, gas nozzle, catcher and skimmer with graphite and adjusting the streaming potential of the liquid (by adjusting the salt concentration and flow rate of the liquid).

The streaming potential of the liquid-microjet was measured using a procedure similar to that reported by Tang *et al.*.[7] A series of 2+1 REMPI measurements of Xe were recorded with the liquid-microjet positioned at various distances from the ionisation point. To accommodate the movement of the jet assembly, the magnet is distanced further from the ionisation point for these streaming potential measurements and so a second TOF-eKE calibration is carried out for this experimental configuration. The eKEs for the Xe ionisation are plotted as a function of distance between the liquid-microjet and the ionisation point (Supplementary Fig. 4) to determine how the eKEs vary with distance from the liquid-microjet, and hence whether the potential is flat in the interaction region.

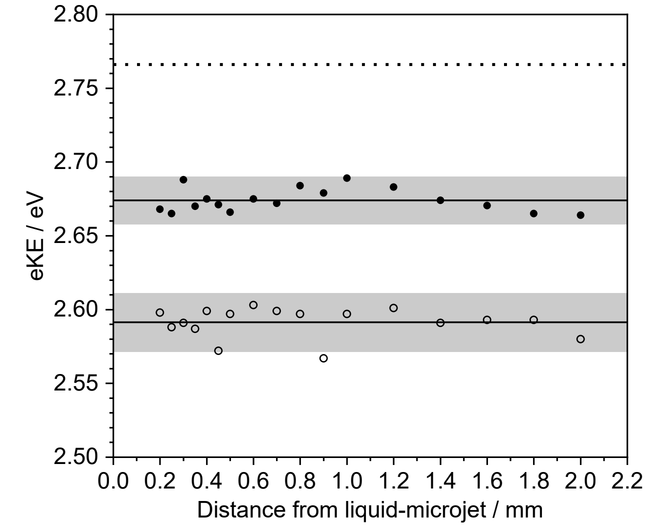

Supplementary Figure 4: Experimentlly measured streaming potentials compared with the expected value of Xe. Experimentally measured eKEs associated with 2+1 REMPI of Xe to the $^2P_{3/2}$ state of Xe^+ at 249.7 nm. Data points show measurements recorded in the presence of aqueous *p*-HBDI$^-$, plotted as a function of the distance between the ionisation point and the liquid-microjet before (filled circles) and after (open circles) recording the photoelectron spectra presented in Fig. 2a of the paper. Solid horizontal lines represent the mean eKEs (2.67 ± 0.03 eV and 2.59 ± 0.04 eV) and shaded areas represent two standard deviations either side of each mean. The dashed line indicates the expected value of 2.77 eV.[5]

This procedure was performed both before and after recording the *p*-HBDI$^-$ spectra. For each

streaming potential measurement, there is little variation in the measured eKEs as a function of the position of the liquid-microjet, showing that the potential is flat (zero streaming potential). The mean eKEs for the measurements taken before and after collecting the p-HBDI$^-$ spectra were 2.67 ± 0.03 eV and 2.59 ± 0.04 eV, respectively (uncertainties are two standard deviations), and are offset from the expected field-free eKE (2.77 eV).[5] The offset between the measured values and the expected field-free values may be attributed to the vacuum level offset between the interaction region and the analyser.[1] The fact that the values are slightly different before and after the collection of the p-HBDI$^-$ spectra could be due to increased water vapour in the interaction region or the catcher or other changes in experimental conditions. To account for the vacuum level offset, we have added the mean difference between mean measured eKEs and expected field-free eKEs (0.1 eV, to 1 s.f.) to give the eKEs that are reported in Table 1 of the paper.

In addition to the uncertainty in the vacuum level offset, additional sources of uncertainty in our measurements include the instrument function for low eKEs (Supplementary Fig. 3) and the effect of inelastic scattering (Supplementary Fig. 6). We account for all these by using error bars for our reported eKEs of ± 0.2 eV.

Gaussian fitting for the 300 nm photoelectron spectrum

The 300 nm spectrum can be fit with one or two Gaussians (Supplementary Fig. 5); however, the residual from the two Gaussian fit suggests that it provides a better description of the processes giving rise to this spectrum.

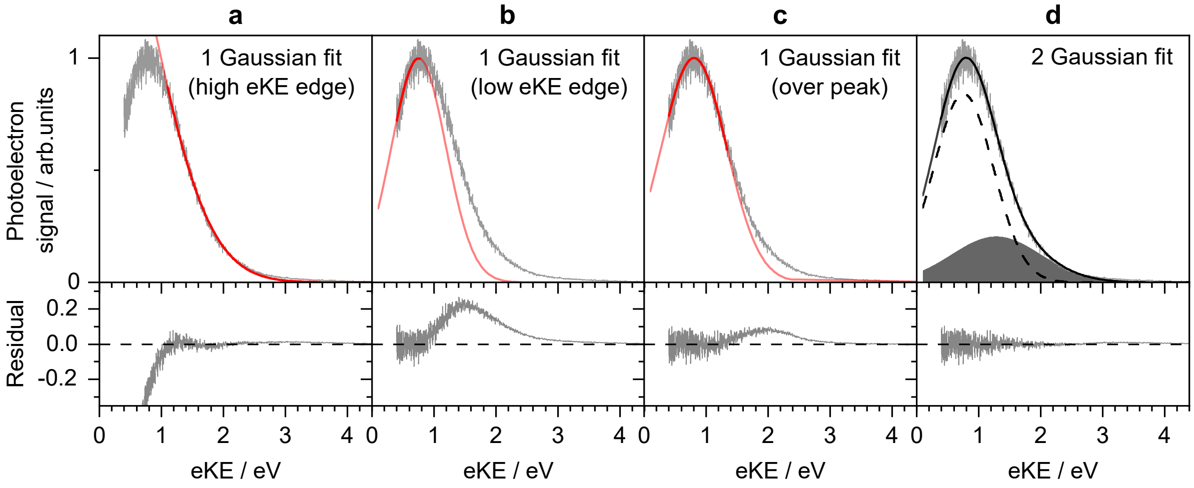

Supplementary Figure 5: Gaussian fitting comparison of the 300 nm MP detachment spectrum of aqueous p-HBDI$^-$. MP detachment photoelectron spectrum of aqueous p-HBDI$^-$ recorded following photoexcitation at 300 nm, plotted as a function of true eKE (after correcting for the vacuum level offset between the interaction region and the analyser and fit with single Gaussians (red lines, **a**-**c**) and two Gaussians (grey shading and dashed line, **d**, as in the main text). Single Gaussians have been fit to the high eKE edge (**a**, peak at -0.1 ± 0.2 eV), low eKE edge (**b**, peak at 0.8 ± 0.2 eV) and in between (**c**, peak at 0.8 ± 0.2 eV). Thick red lines correspond to the regions to which the fits were constrained. The residuals for all fits are shown in the lower panels.

Electron scattering simulation

Before the photoelectrons emerge from the liquid, it is possible that they undergo inelastic collisions with solvent molecules causing a loss of eKE and even loss of electron signal at low eKEs.[8–10] There are various parameters necessary to consider to account for scattering including the penetration depth of the light, the concentration-depth profile of the solute, the initial eKE of the photoelectrons, how likely electrons with such eKE are to lose eKE via various scattering processes and eKE losses associated with such processes. Luckhaus *et al.* used comprehensive Monte-Carlo simulations to model the inelastic scattering contributions to photoelectron spectra of the hydrated electron and extract the true binding energy.[8] Here, we include the details of much simpler one-dimensional simulations to estimate eKE losses we would expect for photoelectrons produced with initial eKEs in the range 0.5-2 eV originating from depths below the surface of the liquid ranging from 1-25 nm. The results are summarised in Supplementary Figure 6 and the simulation details are given below.

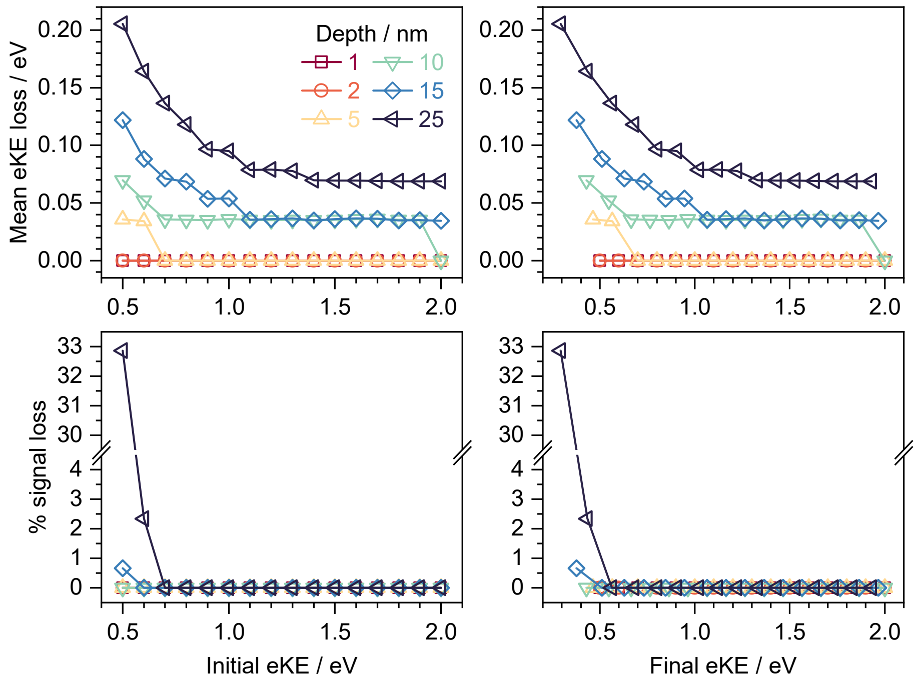

Supplementary Figure 6: Electron scattering simulations. Top: mean eKE losses as a function of initial eKE for electrons starting at different depths in the liquid relative to the surface. Only electrons that emerge successfully from the liquid are included. Bottom: percentage electron signal loss over 10,000 trajectories for different initial eKEs and depths. Data are plotted as a function of initial (left) and final (right) eKE.

Briefly, the electron trajectories were modelled using a random walk of mean free paths estimated using cross-sections taken directly from Luckhaus *et al.*[8] An escape threshold, the minimum eKE required for the electron to escape, of 0.1 eV was employed. eKE losses on collision were sampled randomly from uniform distributions about limits of 0.04 to 0.1 eV for intermolecular scattering events, 0.2 to 0.9 eV for intramolecular scattering events and 5 to 12 eV for electronic scattering events. The inclusion of electronic scattering had almost no effect on the results as only low eKEs were sampled where the cross-sections for electronic transitions are minimal. For each pair of sampling parameters (initial eKE and depth), 10,000 electron trajectories were used to build a distribution.

The depths of 1 to 25 nm were chosen as we expect *p*-HBDI$^-$ concentrations to be higher near the surface due to its low solubility.[11] Moreover, the fact that Gaussian profiles fit effectively with minimal residuals to the experimental data (Supplementary Fig. 2) suggests that inelastic

scattering effects are insignificant and implies photoelectrons are not travelling far within the liquid.

The results of these one-dimensional simulations indicate that photoelectrons generated with eKE > 1 eV near the surface are unlikely to scatter with solvent and will escape with almost no loss of eKE. Photoelectrons generated near the surface with lower eKEs (< 1 eV) are more likely to scatter but the loss of eKE is minimal and within experimental error. Overall, the effectiveness of Gaussian fits to the experimental data and our simulations suggest that scattering effects are minimal for the eKEs measured in this work for a sparingly soluble organic chromophore that has a propensity to reside near the surface, hence justifying our approach to analysis and resulting assignments.

Initial molecular dynamics simulation

The molecular dynamics (MD) simulation system was constructed as a single p-HBDI$^-$ molecule at the centre of a large sphere of water molecules (radius ∼50 Å). The MD simulation was performed within NAMD[12] (developed by the Theoretical and Computational Biophysics Group in the Beckman Institute for Advanced Science and Technology at the University of Illinois at Urbana-Champaign). The CHARMM force field parameters were used to describe the chromophore[13] and the water molecules were described with the TIP3P parameters.[14] The system was initially optimised for 5000 steps. Spherical harmonic boundary conditions with a force constant of 10 kcal/mol·Å^2 were applied during room-temperature MD simulations in the NVT ensemble. The simulations were carried out with an integration step of 1 fs for 2 ns. Then the system was gradually cooled down to 20 K in steps of 1 K during 560 ps. The final MD geometry was then obtained by performing geometry optimisation for an additional 10000 steps.

QM/EFP VDE calculation

The final MD structure was reduced in size by cutting it into smaller spheres with ∼500 (R = 11 Å), ∼1000 (R = 16 Å), and ∼ 1500 (R = 18 Å) water molecules. The quantum-mechanics (QM) region in each case included the chromophore and the six water molecules closest to the chromophore. All other molecules were treated at the effective fragment potential (EFP)[15,16] level. These structures were fully optimized using the PBE0[17,18]/(aug)-cc-pVDZ[19]//EFP approach with diffuse functions only affixed to the oxygen atoms. These calculations and any further calculations were performed within the Firefly software suite[20] (partially based on the GAMESS (US) source code[21]). The optimised structures were used to calculate the VDE employing the energy difference method at the same level of theory. This gives a VDE of 6.0, 6.4 and 6.2 eV for the systems with ∼500, ∼1000, and ∼1500 water molecules, respectively.

QM/EFP/MD VDE calculation

The two systems with ∼1000 and ∼1500 water molecules were used in the subsequent large-scale hybrid QM/EFP/MD calculations to test the convergence of the calculated VDE with respect to the system size. The fully optimised QM/EFP systems were additionally solvated with a large box of water molecules (length ∼100 Å) and MD simulations with periodic boundary conditions were performed with the QM/EFP core kept frozen. The same MD protocol was used as in the initial MD step. Following equilibration, the systems were slowly cooled down, and the final geometry of the outer-shell molecules were optimized with the TIP3P force field parameters.

The final hybrid QM/EFP/MD structures were cut into smaller-sized systems, with distances from water molecules to the chromophore ranging from 10 Å to 45 Å (up to ∼19000 water molecules). The two series with the QM/EFP cores of ∼1000 and ∼1500 water molecules were used to calculate the VDE as a function of the system size. The VDEs were calculated using the energy difference method at the PBE0/(aug)-cc-pVDZ level of theory. The calculated VDEs are shown in Supplementary Fig. 7. The calculated VDE is found to be strongly dependent on the

system size, reaching the converged value of 6.84 eV for a system with ~12500 water molecules ($R = 40$ Å). Importantly, the two series converge to the same VDE.

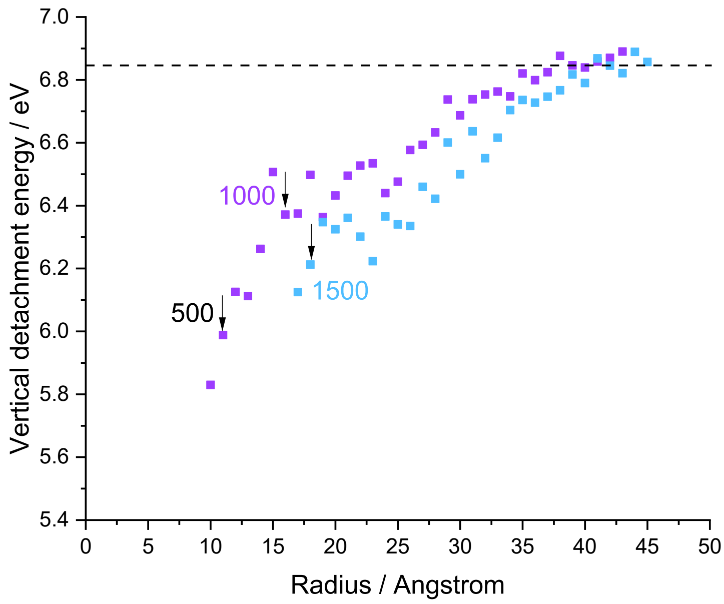

Supplementary Figure 7: Calculated VDEs as a function of the radius of the water sphere used. PBE0/(aug)-cc-pVDZ//EFP calculated VDEs as a function of the radius (R) of the water sphere around the anionic chromophore for the two hybrid QM/EFP/MD systems with the fully optimized QM/EFP cores of ~1000 (purple) and ~1500 (light blue) EFP water molecules. The arrows mark the calculated VDEs of the pure QM/EFP systems with ~500, 1000, and 1500 water molecules.

XMCQDPT2/EFP VDE calculation

Since the calculated VDE is strongly dependent on the system size and reaches the converged value of 6.84 eV at the PBE0/(aug)-cc-pVDZ/EFP level of theory for a system with ~12500 water molecules ($R = 40$ Å), the equilibrium structure of the latter with ~1000 water molecules in the PBE0/EFP core is used in the high-level XMCQDPT2/EFP calculations. The lowest-lying D_0 and D_1 VDEs was calculated using the (aug)-cc-pVDZ basis set, which was augmented by very diffuse functions of the p-type with a 10^{-10} exponent (IP orbitals). The IP functions were placed at a distance of 40 Å from the edge of the water sphere along the direction of the dipole moment of the *p*-HBDI radical (Supplementary Fig. 8). The calculated VDE was found to be insensitive to the position of the IP orbitals outside the water sphere for distances larger than 30 Å. One of these IP orbitals was included in the active space to mimic electron detachment. In addition to the IP orbital, all valence π orbitals, except for the lone pair orbital localised on the nitrogen atom of the imidazolinone ring, were included in the active space, which resulted in 14 electrons distributed over 14 orbitals. The XMCQDPT2 effective Hamiltonian was constructed in the frame of the model space spanned by 10 state-averaged CASSCF(14,14) wavefunctions. The energies of the *p*-HBDI anion in the ground state (S_0) and its radical in the ground (D_0) and first excited (D_1) states with an ejected electron occupying the IP orbital were then obtained in a single XMCQDPT2/SA(10)-CASSCF(14,14)/(aug)-cc-pVDZ+//EFP calculation. The quantum mechanical part included *p*-HBDI$^-$ and six water molecules closest to the chromophore, while all other water molecules were treated using the EFP method. The large-scale XMCQDPT2/EFP calculations gave the S_0–D_0 VDE of 6.71 eV, which was consistent with the value estimated using the energy difference method at the PBE0/EFP level of theory (6.84 eV). The first VDE of 6.7 eV was used for interpreting the experimental results.

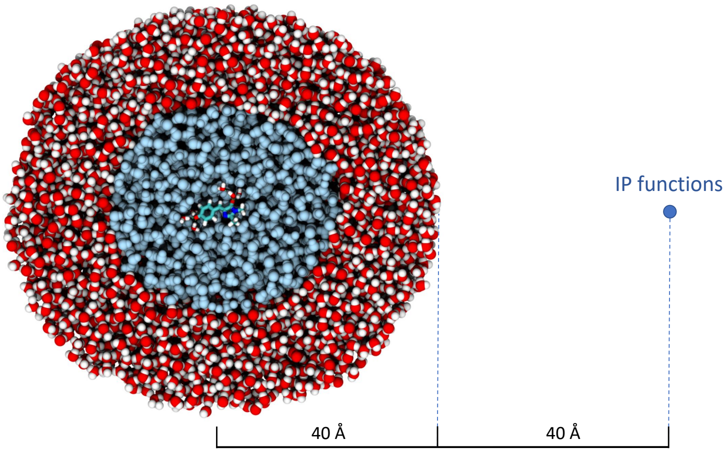

Supplementary Figure 8: Computational geometry for calculating the first VDE. Geometry of the system used to calculate the first VDE at the XMCQDPT2/SA(10)-CASSCF(14,14)/(aug)-cc-pVDZ+/EFPlevel of theory including the extent of the diffuse IP function included within the active space. The structure consists of a QM core (p-HBDI$^-$ and the six closest water molecules) at the centre, an inner shell of optimised EFP water molecules shown in blue (1,496 H_2O extending out to 18 Å from the chromophore) and an outer shell of MD annealed EFP water molecules shown in red/white (11,054 H_2O).

Vertical excitation energy calculation

Vertical excitation energies (VEEs) were calculated for a QM/EFP system with 253 EFP water molecules fully optimized at the PBE0/(aug)-cc-pVDZ//EFP level of theory. The chromophore and six water molecules closest to the chromophore were included in the QM part. The equilibrium geometry is shown in Supplementary Fig. 9.

The S_0–S_n VEEs of the solvated p-HBDI$^-$ anion were calculated using the extended multi-configuration quasi-degenerate perturbation theory, XMCQDPT2,[20,22] combined with the EFP method[23]. The zeroth-order wave functions were constructed using the state-averaged complete active space self-consistent field method, SA-CASSCF, with pure π and mixed n/π active spaces. All valence π orbitals were included in the pure active space, resulting in 16 electrons distributed over 14 orbitals. The π–π^* transitions were calculated using 10 SA(10)-CASSCF(16,14)/(aug)-cc-pVDZ states, which spanned the model space of the XMCQDPT2 effective Hamiltonian. The n–π^* transitions were calculated by adding the n-orbital of the phenolate moiety to the active space, while removing one π orbital, which was represented by a lone pair predominantly localised on the nitrogen atom of the imidazolinone ring. The reduction was based on the analysis of the occupation numbers of the natural orbitals obtained with the pure π active space. For the calculation of the n–π^* transitions, the model space was spanned by two SA(2)-CASSCF(16,14) zeroth-order states, the ground state and the n–π^* target state. The XMCQDPT2 calculations employed a DFT/PBE0-based effective Fock operator to obtain energies of all CASSCF semi-canonical orbitals used in a perturbation theory series. All XMCQDPT2/EFP calculations were performed omitting the fragment polarization term, thus only allowing the QM electron density (both in the ground and excited states) to be polarized in the field produced by effective fragments.

The calculated $\pi\pi^*$ VEE values were also compared to those obtained through the large-scale XMCQDPT2/SA(10)-CASSCF(14,14)/(aug)-cc-pVDZ+//EFP calculations with both valence and detached states included in the state-averaging procedure. The results were in good agreement with each other (see Supplementary Table 1).

The VEEs were also calculated in the solvated neutral radical to estimate the positions of

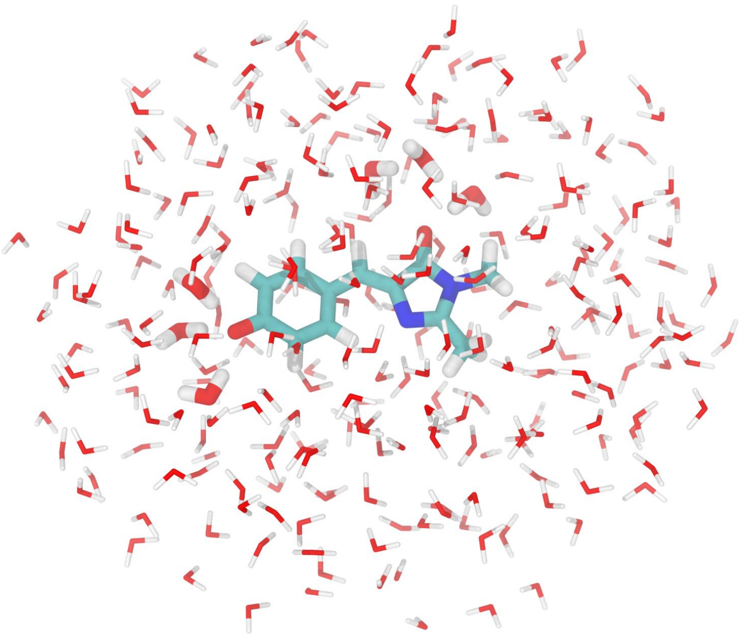

Supplementary Figure 9: PBE0/(aug)-cc-pVDZ//EFP equilibrium geometry of p-HBDI^{-} in water solution used for the VEE calculations. Highlighted is the QM part, which includes the chromophore and six water molecules.

Excited state	$S_1/1\pi\pi^*$	$S_2/2\pi\pi^*$	$S_4/3\pi\pi^*$	$S_5/4\pi\pi^*$	$S_6/5\pi\pi^*$
small	2.79	3.84	4.26	4.51	4.79
large	2.92	3.96	4.21	4.72	4.90

Supplementary Table 1: XMCQDPT2/EFP calculated $\pi\pi^*$ VEEs (eV) using the small system shown in Supplementary Fig. 9 and the large system from Supplementary Fig. 8. In the former case, only valence states are included in the XMCQDPT2/SA(10)-CASSCF(16,14)/(aug)-cc-pVDZ//EFP calculations, while both valence and detached states are considered in the latter case in the XMCQDPT2/SA(10)-CASSCF(14,14)/(aug)-cc-pVDZ+//EFP calculations.

higher-lying detachment thresholds with respect to the ground state of the radical, D_0. The XMCQDPT2/SA-CASSCF(15,14)/(aug)-cc-pVDZ//EFP calculations with pure and mixed active spaces were carried out in the equilibrium geometry of the anion.

Supplementary Note 1

S_0-D_n VDEs and agreement with theory

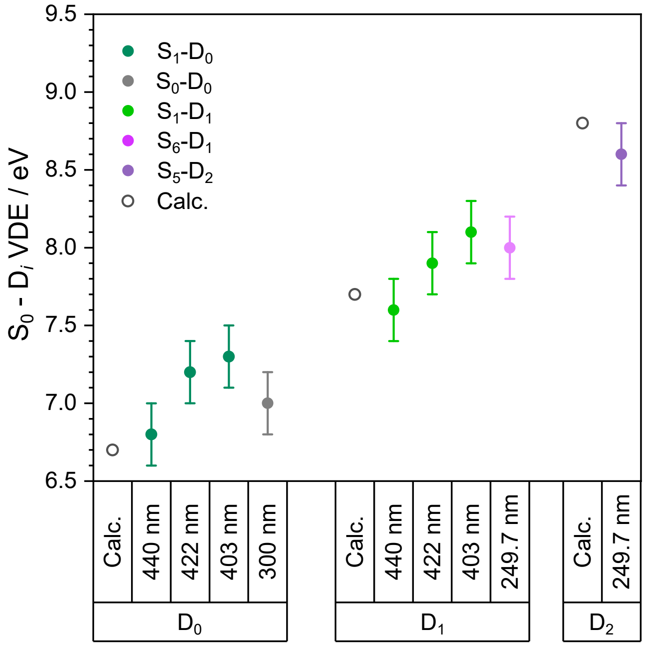

Supplementary Figure 10: Comparison between experimental and calculated VDEs. S_0-D_i VDEs (in eV) determined from photoelectron spectra recorded at the specified wavelengths, as listed in in the main text, and calculated using either a hybrid DFT/EFP/MD approach (S_0-D_0) or with XMCQDPT2/SA(10)-CASSCF(15,14)/(aug)-cc-pVDZ//EFP with respect to the energy of the D_0 state (S_0-$D_{i>0}$). Estimated VDEs obtained using experimental measurements have been coloured to match to the corresponding features in the main text.

Supplementary Note 2

S_0–S_1 absorption profile and adiabatic excitation energy calculation

The S_0/S_1 origin shifts along each normal mode were calculated under the double harmonic parallel-mode approximation, using the XMCQDPT2/SA(2)-CASSCF(14,13)/(aug)-cc-pVDZ//EFP gradient at the Franck-Condon point. The pure π active space was reduced by removing a lone pair of the nitrogen atom of the imidazolinone ring. Vibrational analysis was performed in the ground state using the PBE0/(aug)-cc-pVDZ//EFP method. The fully optimized QM/EFP system with 253 EFP water molecules was used for the calculations (Supplementary Fig. 9). Only the normal modes of the QM part were considered. Based on the calculated origin shifts, the S_0–S_1 vibronic band shape at 300 K was also simulated through direct evaluation of Franck-Condon integrals using our original program VibroniX (Supplementary Fig. 11).

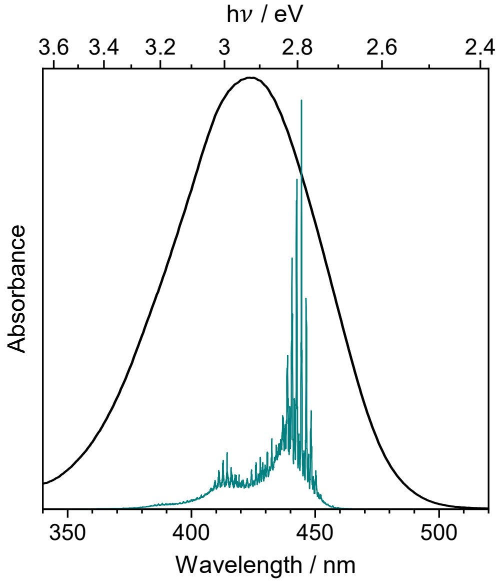

Supplementary Figure 11: Comparison between experimental and calculated absorption profiles. S_0–S_1 calculated absorption profile at 300 K for a single configuration of *p*-HBDI$^-$ in water solution depicted in Supplementary Fig. 9 (green) together with the experimental spectrum (black). The calculated stick spectrum is convoluted with a Gaussian function with a half width at half maximum of 4 cm^{-1}. The most intense transition refers to the 0-0 excitation.

The calculated reorganisation energy in S_1 equals 0.1 eV for a single configuration depicted in Supplementary Fig. 9. Given a very small value of the reorganisation energy of the chromophore and the closest water molecules upon the S_0–S_1 transition, which falls within the experimental error bars, the VEE is used instead of the adiabatic excitation energy (AEE), when interpreting the experimental results. For higher lying excited states, we also assume that VEEs $\approx$ AEEs.

Supplementary References

[1] Nishitani, J., Karashima, S., West, C. W. & Suzuki, T. Surface potential of liquid microjet investigated using extreme ultraviolet photoelectron spectroscopy. *J. Chem. Phys.* **152**, 144503 (2020).

[2] Thürmer, S. *et al.* Accurate vertical ionization energy and work function determinations of liquid water and aqueous solutions (2021). `2104.01630`.

[3] Riley, J. W., Wang, B., Parkes, M. A. & Fielding, H. H. Design and characterization of a recirculating liquid-microjet photoelectron spectrometer for multiphoton ultraviolet photoelectron spectroscopy. *Rev. Sci. Instrum* **90**, 083104 (2019).

[4] Jarvis, G., Evans, M., Ng, C. & Mitsuke, K. Rotational-resolved pulsed field ionization photoelectron study of $NO^+(X^1\sigma^+, v^+ = 0 - 32)$ in the energy range of 9.24–16.80 eV. *J. Chem. Phys.* **111**, 3058 (1999).

[5] Yoshino, K. & Freeman, D. E. Absorption spectrum of xenon in the vacuum-ultraviolet region. *J. Opt. Soc. Am. B* **2**, 1268–1274 (1985).

[6] Perry, C. F. *et al.* Photoelectron spectroscopy of liquid water with tunable extreme-ultraviolet radiation: Effects of electron scattering. *J. Phys. Chem. Lett.* **12**, 2990–2996 (2021).

[7] Tang, Y. *et al.* Time-resolved photoelectron spectroscopy of bulk liquids at ultra-low kinetic energy. *Chem. Phys. Lett.* **494**, 111–116 (2010).

[8] Luckhaus, D., Yamamoto, Y.-i., Suzuki, T. & Signorell, R. Genuine binding energy of the hydrated electron. *Sci. Adv.* **3**, e1603224 (2017).

[9] Malerz, S. *et al.* Low-energy constraints on photoelectron spectra measured from liquid water and aqueous solutions. *Phys. Chem. Chem. Phys.* **23**, 8246 (2021).

[10] Yamamoto, Y. I., Karashima, S., Adachi, S. & Suzuki, T. Wavelength dependence of UV photoemission from solvated electrons in bulk water, methanol, and ethanol. *J. Phys. Chem. A* **120**, 1153–1159 (2016).

[11] West, C. W., Nishitani, J., Higashimura, C. & Suzuki, T. Extreme ultraviolet time-resolved photoelectron spectroscopy of aqueous aniline solution: enhanced surface concentration and pump-induced space charge effect. *Mol. Phys.* **119**, e1748240 (2021).

[12] Phillips, J. C. *et al.* Scalable molecular dynamics with NAMD. *J. Comput. Chem.* **26**, 1781–1802 (2005).

[13] Reuter, N., Lin, H. & Thiel, W. Green fluorescent proteins: Empirical force field for the neutral and deprotonated forms of the chromophore. molecular dynamics simulations of the wild type and S65T mutant. *J. Phys. Chem. B* **106**, 6310–6321 (2002).

[14] Jorgensen, W. L., Chandrasekhar, J., Madura, J. D., Impey, R. W. & Klein, M. L. Comparison of simple potential functions for simulating liquid water. *J. Chem. Phys* **79**, 926–935 (1983).

[15] Gordon, M. S. *et al.* The effective fragment potential method: A QM-based MM approach to modeling environmental effects in chemistry. *J. Phys. Chem. A* **105**, 306–307 (2001).

[16] Gordon, M. S., Fedorov, D. G., Pruitt, S. R. & Slipchenko, L. V. Fragmentation methods: A route to accurate calculations on large systems. *Chem. Rev.* **112**, 632–672 (2012).

[17] Perdew, J. P., Burke, K. & Ernzerhof, M. Generalized gradient approximation made simple. *Phys. Rev. Lett.* **77**, 3865–3868 (1996).

[18] Adamo, C. & Barone, V. Toward reliable density functional methods without adjustable parameters: The PBE0 model. *J. Chem. Phys.* **110**, 6158–6170 (1999).

[19] Kendall, R. A., Dunning, T. H. & Harrison, R. J. Electron affinities of the first-row atoms revisited. systematic basis sets and wave functions. *J. Chem. Phys.* **96**, 6796–6806 (1992).

[20] Granovsky, A. A., Firefly version 8.2.0, http://classic.chem.msu.su/gran/firefly/index.html.

[21] Schmidt, M. W. *et al.* General atomic and molecular electronic structure system. *J. Comput. Chem.* **14**, 1347–1363 (1993).

[22] Granovsky, A. A. Extended multi-configuration quasi-degenerate perturbation theory: The new approach to multi-state multi-reference perturbation theory. *J. Chem. Phys.* **134**, 214113 (2011).

[23] Day, P. N. *et al.* An effective fragment method for modeling solvent effects in quantum mechanical calculations. *J. Chem. Phys.* **105**, 1968–1986 (1996).
